# Aerobic exercise improves cognitive impairment in mice with type 2 diabetes by regulating the MALAT1/miR-382-3p/BDNF signaling pathway in serum-exosomes

**DOI:** 10.1186/s10020-023-00727-1

**Published:** 2023-09-22

**Authors:** Mingzhu Wang, Kangling Xie, Shengnan Zhao, Nan Jia, Yujiao Zong, Wenping Gu, Ying Cai

**Affiliations:** 1 National Clinical Research Center for Geriatric Disorders, Department of Rehabilitation, Xiangya Hospital Central South University, Changsha, 410008 Hunan China; 2 National Clinical Research Center for Geriatric Disorders, Department of Neurology, Xiangya Hospital Central South University, Changsha, 410008 China

**Keywords:** Aerobic exercise, Serum-exosomes, Non-coding RNA, Type 2 diabetes, Hippocampal neurons, MALAT1, miR-382-3p, BDNF

## Abstract

**Background:**

It has been documented that aerobic exercise (AE) has a positive effect on improving cognitive function in type 2 diabetes (T2DM) patients. Here, we tried to explore how AE regulates the expression of long non-coding RNA in serum-exosomes (Exos), thereby affecting cognitive impairment in T2DM mice as well as its potential molecular mechanism.

**Methods:**

T2DM mouse models were constructed, and serum-Exos were isolated for whole transcriptome sequencing to screen differentially expressed lncRNA and mRNA, followed by prediction of downstream target genes. The binding ability of miR-382-3p with a long non-coding RNA MALAT1 and brain-derived neurotrophic factor (BDNF) was explored. Then, primary mouse hippocampal neurons were collected for in vitro mechanism verification, as evidenced by the detection of hippocampal neurons' vitality, proliferation, and apoptosis capabilities, and insulin resistance. Finally, in vivo mechanism verification was performed to assess the effect of AE on insulin resistance and cognitive disorder.

**Results:**

Transcriptome sequencing analysis showed that MALAT1 was lowly expressed and miR-382-3p was highly expressed in serum-Exos samples of T2DM mice. There were targeted binding sites between MALAT1 and miR-382-3p and between miR-382-3p and BDNF. In vitro experiments showed that MALAT1 upregulated BDNF expression by inhibiting miR-382-3p. Silencing MALAT1 or overexpressing miR-382-3p could reduce the expression of INSR, IRS-1, IRS-2, PI3K/AKT, and Ras/MAPK, inhibit neuronal proliferation, and promote apoptosis. In vivo experiments further confirmed that AE could increase the expression of MALAT1 in serum-Exos to competitively inhibit miR-382-3p and upregulate BDNF expression, thereby improving cognitive impairment in T2DM mice.

**Conclusion:**

AE may upregulate the expression of MALAT1 in serum-Exos to competitively inhibit miR-382-3p and upregulate BDNF expression, thus improving cognitive impairment in T2DM mice.

**Supplementary Information:**

The online version contains supplementary material available at 10.1186/s10020-023-00727-1.

## Introduction

Type 2 diabetes mellitus (T2DM) is a chronic metabolic disorder characterized by high blood sugar levels and decreased insulin secretion and sensitivity (Ho et al. 2020). T2DM is on the rise worldwide, with increasing prevalence in all regions, especially in low-income and middle-income countries. This surge can be primarily attributed to epidemiological shifts brought about by changes in nutrition, urbanization, and sedentary lifestyles (Tinajero and Malik 2021). T2DM is not only associated with an increased risk of cardiovascular disease but may also lead to cognitive decline and dementia (van Sloten et al. 2020).

Obesity is an important risk factor for the development of T2DM (Malone and Hansen 2019). Obesity can lead to insulin resistance and affect insulin secretion and utilization (Amin et al. 2019) As obesity progresses, fatty tissue begins to secrete hormones such as tumor necrosis factor-alpha (TNF-α) and interleukin-6 (IL-6), which exacerbate insulin resistance (Rohm et al. 2022). Furthermore, adipocytes secrete a substance called adipokines, which inhibit the synthesis and secretion of insulin in pancreatic cells (Dufau et al. 2021).

A large body of research has shown a correlation between insulin resistance and cognitive impairment, and multiple mechanisms may explain this association (Barber et al. 2021; Cui et al. 2022). In insulin resistance, the body requires more insulin to maintain blood sugar levels, depriving brain neurons of sufficient energy support. As a result, it can cause direct damage to brain function and affect cognitive ability (Flores-Dorantes et al. 2020). In addition, insulin may also affect cognitive function through neural inflammation, oxidative stress, and neuronal apoptosis (Michailidis et al. 2022).

Recent studies have shown that non-coding RNA (ncRNA) in serum exosomes has the potential to be used for disease diagnosis and treatment (Mugoni et al. 2022). In addition, many cell types secrete exosomes and contain various bioactive molecules, including ncRNA, proteins, and lipids (Isaac et al. 2021; Wang et al. 2022a, b; Wang et al. 2022a, b). Importantly, evidence suggests that exosomes and ncRNA may play important roles in the pathogenesis of T2DM and cognitive impairment (Cao et al. 2022).

Exercise training and physical activity have been considered cornerstones for the prevention and treatment of T2DM (Pan et al. 2018). Aerobic exercise (AE) has been shown to positively affect cognitive function in T2DM patients (Magnon et al. 2022). Our previous study has reported that exercise is able to downregulate MALAT1 to reduce resistin and can increase microRNA-382-3p (miR-382-3p) expression in the serum of insulin resistance mice (Liu et al. 2019) AE is associated with increased brain-derived neurotrophic factor (BDNF) in the hippocampus of diabetic rats (Gaitan et al. 2021).

From all the above, we tried to explore how AE affects the MALAT1/miR-382-3p/BDNF signaling pathway in serum-Exos to improve cognitive impairment related to T2DM. This study will provide a better understanding of the potential mechanisms of cognitive impairment related to T2DM and will provide new therapeutic strategies for managing cognitive impairment in T2DM patients.

## Materials and methods

### Construction of T2DM mouse model

One hundred eight-week-old healthy male C57BL/6J mice, purchased from Beijing Vital River Laboratory Animal Technology Co. Ltd. (219, Beijing, China), were housed in SPF conditions with standard feed and water, and maintained at a controlled temperature of 23–26 °C, with a humidity of 60% and a 12-h light/dark cycle. After 1 week of acclimatization, mice in the HF group were fed with a 45% Kcal HFD (D12451, Shanghai FBSH Biotechnology, China) for 12 weeks and injected with 100 mg/kg streptozotocin (STZ, S0130, Sigma-Aldrich, USA, dissolved in citrate buffer) at the fourth week into the abdominal cavity to induce diabetes. Mice in the blank group were fed with a normal diet (D12450J, Shanghai FBSH Biotechnology, China) for 12 weeks and injected with the same volume of citrate buffer (P4809, Sigma-Aldrich, USA, STZ solvent) at the fourth week into the abdominal cavity. After continuing their respective diets for 12 weeks, the mice were weighed and fasting blood glucose (FBG) and insulin (FIN) levels were measured. For overnight-fasted mice, the serum was separated following centrifugation at 1000 rpm for 10 min. The FBG level of the mice was measured using a RuiTe GM300 glucometer (100145, Beijing Glucometer Website, China). The FIN levels in the blood and cerebrospinal fluid (CSF) of the mice were measured using a mouse insulin ELISA assay kit (JL11459, Shanghai Jianglai Biotechnology, China). Insulin resistance index (HOMA-IR) = (FBG × FIN)/22.5, 10 mice/per group. In addition, we performed glucose tolerance (GTT) and insulin resistance (ITT) tests to verify the successful construction of the T2DM mouse model (Li et al. 2017).

### Transcriptome sequencing analysis

We used whole transcriptome sequencing to screen for differentially expressed lncRNA and mRNA in T2DM and blank mouse serum Exos. After 12 weeks of high-fat and high-sugar diet feeding, 10 T2DM mice and 10 blank mice were selected. The mice were placed in a closed euthanasia chamber (RC-100, Shanghai Yuyan Instruments Co., Ltd., China) with a CO_2_ ventilation rate of 30% of the chamber volume per minute to ensure a complete loss of consciousness in the mice. Then, their serum exosomes were collected for sequencing analysis. Firstly, serum exosomes were isolated by centrifugation (the specific methods were seen in Isolation and identification of Exos section Aguiar et al. 2018; Liu et al. 2018), and then total RNA was extracted from serum exosomes using a total RNA extraction kit for quality control and purification.

The Illumina NextSeq 500 platform was used for whole transcriptome sequencing, library establishment, and sequencing. First, the raw data obtained from sequencing were quality controlled by removing low-quality sequences, adapters, and low-quality reads to obtain high-quality clean reads. Then, HISAT2 software was used to align clean reads to the mouse genome with the alignment results and Unmapped reads obtained. Subsequently, the StringTie tool was used to merge the alignment results of all samples and construct a transcriptome assembly file for the entire sample. Finally, edgeR software was used to analyze differentially expressed genes among groups and perform GO functional and enrichment analysis.

According to the results of whole transcriptome sequencing, differentially expressed mRNA and lncRNA were selected for RT-qPCR validation. Firstly, primers were designed based on the sequences obtained from whole transcriptome sequencing, and then samples were subjected to reverse transcription and qPCR analysis. Finally, the accuracy of the analysis results was verified by comparing RT-qPCR results with whole transcriptome sequencing results. In addition, transcriptome sequencing analysis was conducted for differentially expressed mRNA and lncRNA, including functional enrichment analysis of differentially expressed genes and construction of differentially expressed gene networks to discover their roles in T2DM pathogenesis.

### Grouping of mice intervention

The mice were divided into 10 groups, each with 10 mice. The specific treatment is displayed in Additional file 3: Table S1.

During the initial stage of the high-fat diet, a part of the mice in the HF group underwent running training (HF + AE group). The mice underwent adaptive training for 5 days in the first week, running for 10 min each day at a speed of 10 m per minute. By the fifth day, the exercise intensity gradually increased to 60% of the maximum power measured in the increasing load test on the mouse. It was maintained at a treadmill inclination angle of 0 degrees. The training period was 12 weeks, 3 times per week, and the duration increased by 10 min each week. By the sixth week, the total training time per session reached 60 min. None were treated with insulin injections. After 12 weeks, serum was collected to isolate Exos.

For mice requiring an injection of Exos, silent virus, or agomir, they were first anesthetized by intraperitoneal injection of 50 mg/kg pentobarbital sodium (P3761, Sigma-Aldrich, USA) and then placed on a new standard™ brain stereotaxic instrument (51,503, Beijing Shilian Bo Research Technology Co., Ltd., China) and shaved. The lateral ventricle was located (relative to the bregma stereotaxic coordinates: 0.4 mm AP, ± 1 mm ML, -1.5 mm DV). A 29-gauge needle was used in conjunction with a 0.38 mm polyethylene tube and a 25 μL Hamilton syringe. The needle was clamped and exposed to 4.5 mm, and the injection speed was 0.5 μL/min. Each side was injected with 3 μL (3 × 10^9^ Exos) (Micci et al. 2019), 3 μL of virus diluted to 1 × 10^9^ TU/mL (Zhou et al. 2018a, b; Rolfes et al. 2020), or 3 μL of atomic-NC or miR-382-3p agomir (0.8 nmol dissolved in 3 μL PBS) (Zuo et al. 2019).

After injection, the needle was kept in place for 5 min and removed, the skull was sealed with bone wax, and then the scalp was sutured and disinfected. Next, the mice were placed on a heated pad for recovery, and once they regained consciousness, serum and hippocampal tissue were collected to determine whether Exos could be transferred to the brain parenchyma. Then, the brains of mice were collected after a 5-h intracerebroventricular injection of PKH-67-labeled Exos, and the mice were placed in a closable euthanasia device (RC-100, Shanghai Yuyan Scientific Instrument Co., Ltd. China), the CO_2_ ventilation rate of 30% of the chamber volume per minute to ensure complete loss of consciousness in the mice, and then serum and hippocampal tissue were collected to prepare fresh brain slices and examine the PKH-67-Exos in the brain parenchyma and hippocampus with a confocal microscope (Cui et al. 2018; Micci et al. 2019; Wang and Yang 2021).

The core plasmid (PLKO.1) and the accessory plasmid (RRE, REV, Vsvg) were used for packaging the virus, and the silencing sequence of the target gene was inserted into the mouse. The lentivirus was purchased from Shanghai HANBIO Technology Co., Ltd. (Shanghai, China), and the primer sequence and plasmid construction are detailed in Additional file 3: Table S2. Agomir-NC and miR-382-3p agomir were purchased from Guangzhou Ruibo Biological Technology Co., Ltd. (Guangzhou, Guangdong, China). The Exos inhibitor GW4869 was purchased from MED Chem Express, dissolved in DMSO, and injected in mice intraperitoneally with 2.5 μg/g GW4869 (Essandoh et al. 2015), twice a day (Koide et al. 2023). The experimental procedures and animal use plan were approved by the Ethics Committee of our institution (approval number: CSU-2022-0001-0055).

### Morris Water Maze test

Cognitive function assessment of mice was performed using the Morris Water Maze (MWM) experiment using a circular water tank with a diameter of 120 cm filled with tap water at 22 ± 2 ℃. A platform board submerged 1 cm below the water surface (76–0021, Panlab, Spain) was placed inside the tank. The training and testing duration lasted 60 s. During the training period, each mouse was placed in one quadrant of the pool facing the wall. If a mouse failed to reach the platform within 60 s, it was guided to the platform and allowed to stay there for 15 s. During the testing period, the platform was removed, and the mice were given 60 s to search for the platform, which lasted for six consecutive days. The Smart 3.0 video tracking system (Panlab, Spain) was used to record and analyze the mice's swimming path in the maze (Lu et al. 2020).

### Y Maze experiment

The Y Maze consists of three arms (one start arm and two target arms) sized at 30 × 10 × 20 cm, interconnected by cross-connections (76-0079, Panlab, Spain). The training and testing duration lasted for 2 min. In the training phase, mice underwent ten trials per day with at least 20 min intervals between the trials, which lasted for four consecutive days. The selection of each target arm should be the same, and the spontaneous alternation percentage was calculated (Lu et al. 2020).

### Isolation and identification of Exos

Blood samples were collected from mice fed with a normal diet, mice fed with a high-fat diet, and mice subjected to exercise training after a high-fat diet, and then centrifuged at 10,000*g* for 30 min and at a speed of 100,000×*g* for 90 min using a Sorvall MTX150 high-speed centrifuge (46,963, Thermo Fisher Scientific, USA). The samples were resuspended in 1 × PBS, treated with 25 mL of cold 1 × PBS, centrifuged again at 100,000×*g* for 70 min, resuspended in 100 µL of 1 × PBS, and stored immediately at 80 ℃ (Aguiar et al. 2018; Liu et al. 2018).

Transmission electron microscopy (TEM) was used to identify Exos. The Glaciostem transmission electron microscope (GLACIOSTEM, Thermo Fisher Scientific, USA) was adopted for observing and photographing.

Dynamic light scattering (DLS) was used to measure the diameter of Exos particles: the Exos sample was diluted to a ratio of 1:50 and the Zetasizer Ultra instrument (at Malvern Panalytical, UK) was used to detect the diameter of Exo when excited with a light wavelength of λ = 532 nm in a mixture of 0.15 M NaCl.

The BCA protein analysis kit (P0011, Shanghai Beyotime Biotechnology, China) was used to measure the protein concentration in Exos particles. Then, Western blot was applied to detect the expression of Exos-related proteins such as CD9, CD63, and TSG101.

### Exos tracing

The PKH67 green fluorescent cell linker enzyme Mini kit (Mini67, Sigma-Aldrich, USA) was employed to stain the Exos membrane. In short, Exos and the stain were mixed and incubated at 37 °C for 15 min, followed by centrifugation (120,000×*g*) for 90 min using ultracentrifugation. After removal of the unstained dye and twice washing with PBS, the sample was ultracentrifuged (120,000×*g*) again for 90 min. Before use, the marked Exos were resuspended in PBS. The cell nucleus was stained with DAPI (D9542, Sigma-Aldrich, USA) for 5 min. Fluorescence signals were detected by confocal laser microscopy (FV3000, Olympus, Japan) to determine whether the added Exos fused with the cells.

### Glucose tolerance test (GTT) and insulin resistance test (ITT)

After 12 weeks of dietary intervention for GTT, mice were fasted for 16 h and then injected with glucose intraperitoneally at a dose of 1.75 g/kg body weight. For ITT, mice were fasted for 6 h and then injected with 1 IU/kg human insulin (I2643, Sigma-Aldrich, USA) intraperitoneally. Blood glucose levels of mice were measured using the GM300 blood glucose meter at 0, 30, 60, 90, and 120 min (Li et al. 2018; Cai et al. 2021).

### TUNEL staining

The experiment was performed using the apoptosis detection kit (C1098, Shanghai Beyotime Biotechnology, China). The sample was dewaxed in xylene for 5 min, dewaxed with fresh xylene for 5 min, and then treated with anhydrous ethanol for 5 min, 90% ethanol for 2 min, 70% ethanol for 2 min, and distilled water for 2 min. Then, the sample was incubated with proteinase K containing 20 μg/mL without DNase at 37 °C for 15 min. After washing with PBS 3 times, the sample was incubated with the TUNEL detection solution at 37 °C in the dark for 60 min. After that, the sample was treated using DAPI mounting medium. The apoptosis rate of cells was observed, photographed, and counted under a microscope (catalog number: BX63, Olympus, Japan).

### BF-188 staining

BF-188 (025-18801, Whatman, UK) was dissolved in 100% ethanol to a final concentration of 200 μM and then diluted with distilled water to prepare a 100 μM solution of the compound. Mouse brain sections were dewaxed, treated with the compound solution for 10 min, washed with PBS, and sealed with a fluorescent fading inhibitor. Finally, the results were observed, photographed, and recorded using a microscope (BX63, Olympus, Japan).

### Primary mouse neuron cell culture and infection

C57BL/6J mice (219, VitalRiver, Beijing, China) on postnatal day 1 were selected and placed in Hank's balanced salt solution (14170112, Gibco, USA) containing 1 mM sodium pyruvate (11360070, Gibco, USA) and 10 mM HEPES (15630080, Gibco, USA), without Ca^2+^ and Mg^2+^. The hippocampal tissue was separated in HBSS solution containing 0.125% trypsin (Catalogue No: 25200056, Gibco, USA) for 10 min, and the tissues were dispersed into single cells by triturating using a Pasteur pipette. The digestion was terminated by using DMEM containing 10% FBS (12491015, Gibco, USA). The tissues were allowed to stand for 3 min and centrifuged at 2000 rpm for 2 min. The precipitate was cultured in Neurobasal medium (21103049, Gibco, USA) containing B-27 (17504044, Gibco, USA), 0.5 mM l-glutamine (25030081, Gibco, USA), and 1% penicillin–streptomycin (15070063, Gibco, USA). These cells were seeded at a density of 4 × 10^6^ in 6-well dishes coated with PDL (100 μg/mL) (A3890401, Gibco, USA). Half of the culture medium without glutamine was replaced every 2–3 days with fresh medium. The purity of the neurons was about 95%, and the neurons were cultured for 7 days before subsequent experiments (Hu et al. 2012). The cells in the corresponding groups were treated for 48 h, and the collected cells were analyzed in the subsequent experiments. The high glucose group (HG group) was cultured in a medium containing 45 mmoL/L glucose (Sun et al. 2018).

For HEK293T cell culture, HEK293T cells (CC-Y1010, Shanghai Enzyme-linked Biotechnology Co., Ltd., China) were cultured in DMEM medium containing 10% fetal bovine serum and 1% penicillin–streptomycin (15070063, Gibco, USA) (Balogh et al. 2019). The cells were cultured at 37 °C in a humidified incubator with 5% CO_2_. Cell grouping is displayed in Additional file 3: Table S3.

Lentiviruses were constructed using core plasmids (PLKO.1) and auxiliary plasmids (RRE, REV, Vsvg) containing silencing target gene sequences or cDNA sequences. The cells were treated with a virus solution with a titer of 1 × 10^9^ TU/mL for 6 h, and then the medium was changed for further culturing. In addition, neurons were treated with 20 μM GW4869 for 30 min. The lentivirus was purchased from Shanghai HANBIO Co., Ltd. (Shanghai, China), and the company provided the primer sequences and plasmid construction (Additional file 3: Table S2). In addition, miR-382-3p mimic and miR-382-3p inhibitor were purchased from RiboBio (miR10004691-1-5, miR20004691-1-5).

### Stable cell line screening

Cells were plated in 24-well plates at a density of 5 × 10^4^ cells/well and incubated overnight at 37 ℃. The next day, the cells were incubated with fresh media containing different concentrations of puromycin (including 1 μg/mL, 2.5 μg/mL, 5 μg/mL, and 10 μg/mL) (60210ES25, YEASEN). The fresh medium was changed every two days and the cell growth was observed daily to determine the minimum drug concentration of 5 μg/mL that effectively kills non-infected cells within 4–6 days.

### RT-qPCR

Total RNA was extracted using Trizol (16096020). mRNA was prepared using the cDNA first-strand synthesis kit (D7168L, Beyotime), and miRNA was prepared using the miRNA first-strand cDNA synthesis kit with poly(A) tailing (B532451, Shanghai Sangon Biotech). cDNA synthesis was performed following the related instructions.

According to the kit instructions, the RT-qPCR kit (Q511-02, Nanjing Vazyme Biotech) was selected for experiments.

In this experiment, MALAT1 was normalized to β-actin, while miR-382-3p was normalized to U6. Shanghai Sangon Biotech provided the primer sequences (Additional file 3: Table S4). The 2^−ΔΔCt^ method was used to calculate the fold change in the expression of the target gene between the experimental and control groups, where ΔCt represents Ct (target gene) − Ct (internal reference), and ΔΔCt = ΔCt _experimental group_ − ΔCt _control group_.

### Western blot

The total protein was extracted from tissues and cells using RIPA lysis buffer (P0013B, Beyotime) containing 1 mM PMSF, following the kit's instructions (P0028, Beyotime). The total protein concentration of each sample was determined using the BCA kit (P0011, Beyotime).

Following electrophoresis separation, the protein in the gel was transferred onto a PVDF membrane (1620177, BIO-RAD, USA) and blocked with 5% skim milk powder or 5% BSA for 1 h at room temperature. In addition, the following primary antibodies (Additional file 3: Table S5) were added to the membrane and incubated overnight.

The next day, the membrane was incubated with HRP-labeled goat anti-rabbit IgG secondary antibody (ab6721, 1:5000, Abcam, UK) at room temperature for 1 h. Finally, the membrane was washed thrice with 1 × TBST buffer at room temperature for 5 min each time. The protein bands were exposed using an Image Quant LAS 4000C gel imaging system (GE Company, USA). β-actin was used as an internal reference protein of the cells, and the ratio of the grayscale value of the target band to the reference band was used as the relative expression of the protein to detect the expression of each protein.

### CCK-8 assay

Cell proliferation was detected using the Cell Counting Kit-8 (CCK-8) assay kit (C0037, Beyotime). After different treatments, cells were incubated with 10 μL of CCK-8 solution at 0 h, 24 h, 48 h, and 72 h and then incubated for 1 h in a cell culture incubator. The absorbance was measured at 450 nm using a multifunctional microplate reader Varioskan LUX, and the cell proliferation curve was plotted (Zhan et al. 2018).

### EdU staining

Cell proliferation was detected using the EdU Cell Proliferation Assay Kit (C0075S, Beyotime). After different treatments, cells were incubated with a medium containing 10 μM EdU at 37 °C for 2 h. After removing the culture medium, the cells were fixed with 1 mL of 4% paraformaldehyde at room temperature for 15 min, washed thrice with 1 mL of PBS per well (each for 3 min), and treated with 1 mL of PBS containing 0.3% Triton X-100 at room temperature for 15 min for membrane permeabilization. After removing the permeabilization solution, cells were washed with PBS and stained with Hoechst 33342 for 10 min. Images were observed and photographed under a microscope (BX63, Olympus, Japan). Five random fields were selected for each sample to record the EdU-positive cell rate.

### Flow cytometry

After different treatments, cells were digested and collected in a flow tube. The cells were washed thrice with cold PBS, resuspended in 200 μL buffer, and incubated with 10 μL of Annexin V-FITC and 5 μL of PI for 15 min at room temperature under light avoidance conditions. The reaction solution was added with 300 μL buffer. Cell apoptosis was detected using the Attune NxT flow cytometer (Thermo Fisher Scientific, USA). In addition, the percentage of cells in the Q2 and Q3 quadrants was recorded (Fang et al. 2017).

### Dual-luciferase reporter gene experiments

MALAT1 gene fragments (MALAT1 Wt) and mutant fragments (MALAT1 Mut) that bind to miR-382-3p, as well as BDNF mRNA 3'UTR gene fragments (BDNF Wt) and mutant fragments (BDNF Mut) were synthesized and inserted into the pMIR-REPORT vector (AM5795, Thermo Fisher Science, USA). After restriction enzyme digestion, the target fragment was inserted into the vector using T4 DNA ligase (M0204S, New England Biolabs, USA). Lipofectamine 3000 (L3000001, Thermo Fisher Science, USA) transfection reagent was used to co-transfect 300 ng plasmid with either 50 nM mimic-NC or miR-137 mimic into HEK293T cells. The cells were then incubated at 37 ℃ in a humidified incubator containing 5% CO_2_ for 48 h, collected, and lysed. The Dual-Luciferase® Reporter Assay System kit (E1910, Promega, USA) was used to measure luciferase activity using the GloMax® 20/20 Luminometer (E5311, Promega, USA). All vectors were constructed by Shanghai Sangon Biotech Co., Ltd.

### RIP experiment

The RIP kit (RIP-12RXN, Sigma-Aldrich, USA) was adopted to detect the binding between MALAT1, miR-382-3p, BDNF, and AGO2 protein. Upon reaching 80–90% confluency, the culture medium was removed. Then, the cells were lyzed using an equal volume of RIPA lysis buffer (P0013B, Beyotime) for 5 min and centrifuged at 14,000 rpm for 10 min at 4 ℃, with the supernatant collected. A portion of the cell extract was used as input, and the rest was incubated with antibodies for co-precipitation (Zhang et al. 2020). The RIP antibody we used was AGO2 (1:100, ab186733, Abcam, UK) and IgG (1:100, ab200699, Abcam, UK, negative control).

### Statistical analysis

SPSS software (version 21.0, IBM, USA) was selected to perform statistical analysis of the data in this study. The measurement data were expressed as mean ± standard deviation, and normality and homogeneity of variance were tested first. When the data met the normal distribution and variance homogeneity conditions, a non-paired t-test was used for comparison between groups, one-way analysis of variance or analysis of variance for repeated measures for comparison among multiple groups, and post hoc analysis was performed using Tukey's method. A P value less than 0.05 indicated statistical significance.

## Results

### AE can alleviate cognitive impairment in T2DM mice

**Fig. 1:**
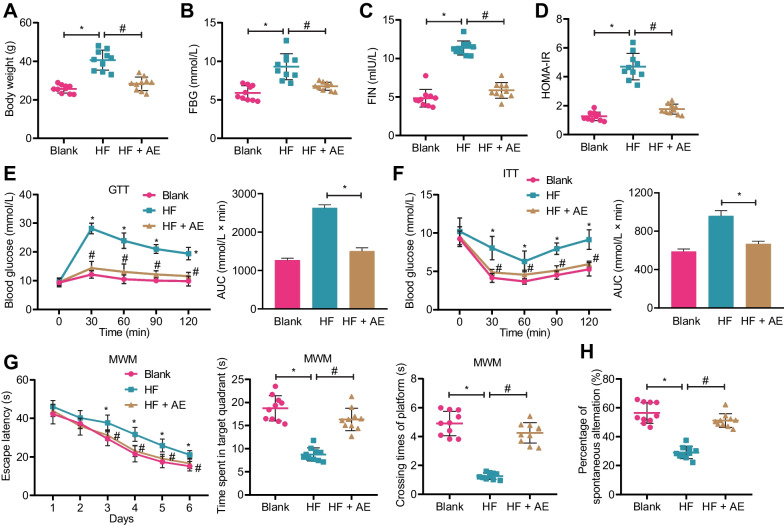
Effects of AE on cognitive impairment in T2DM mice. **A** Changes in body weight of mice in each group; **B** ELISA detection of fasting blood glucose levels in the serum of mice in each group; **C** ELISA detection of fasting serum insulin levels in mice in each group; **D** Comparison of insulin resistance index in mice in each group; **E** GTT and GTT AUC in mice in each group; **F** ITT and ITT AUC in mice in each group; **G** Water maze experiment was used to test the escape latency within 6 days, time spent in the target quadrant, and time to cross the target platform of mice in each group; **H** Y maze experiment was used to test the spontaneous alternation percentage of mice in each group; n = 10, *P < 0.05 compared with blank group, ^#^P < 0.05 compared with HF group

A previous study has shown that AE can improve cognitive impairment in T2DM mice (Callisaya and Nosaka 2017). Therefore, we conducted in vivo experiments to verify this finding. First, we verified whether the T2DM mouse model was successfully established. Compared with the blank group, mice in the HF group showed significantly increased body weight, FPG levels, FIN levels, and HOMA-IR, while the body weight, FPG, FIN, and HOMA-IR decreased significantly in mice in the HF + AE group (Fig. 1A–D).

In the GTT, after the intraperitoneal injection of glucose for 30 min, the blood glucose levels in all three groups of mice peaked and then gradually declined. Compared with the blank group, mice in the HF group showed an overall increase in blood glucose and increased GTT AUC, indicating impaired glucose tolerance, while the blood glucose and GTT AUC of mice in the HF + AE group decreased, suggesting improved glucose tolerance (Fig. 1E). The ITT showed that after insulin injection 30 min, the blood glucose of all three groups of mice showed a valley and then slowly increased. Compared with the blank group, mice in the HF group had overall increased blood glucose and increased ITT AUC, indicating insulin resistance, while the blood glucose and ITT AUC in the HF + AE group decreased, suggesting improved insulin resistance (Fig. 1F).

In the water and Y maze experiments, the results showed that compared with the blank group, in the platform searching experiment, the escape latency time of mice in the HF group significantly increased, the time spent in the target quadrant, platform crossings, and spontaneous alternation percentage were significantly reduced. Compared with the HF group, mice in the HF + AE group showed a significant decrease in the escape latency time and a significant increase in the time spent in the target quadrant, platform crossings, and spontaneous alternation percentage during the platform searching experiment (Fig. 1G, H).

**Fig. 2:**
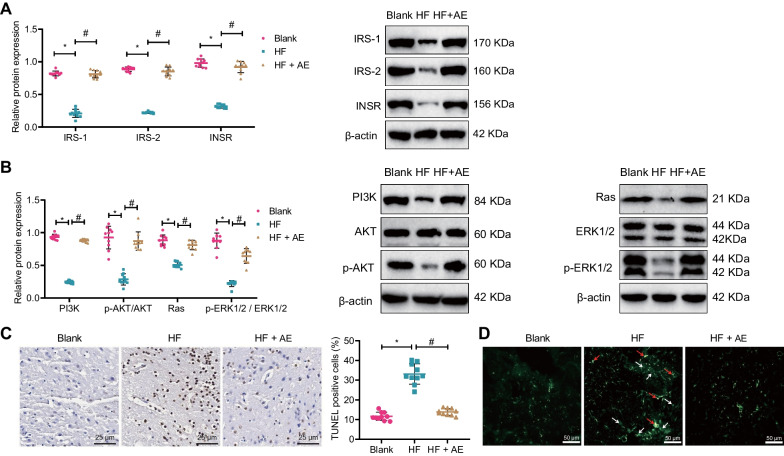
Effects of AE on protein expression and neuronal apoptosis in the hippocampus of T2DM mice. **A** Western blot detection of INSR, IRS-1, and IRS-2 protein expression in the hippocampus of mice in each group; **B** Western blot detection of PI3K, AKT, p-AKT, Ras, Erk1/2, and p-Erk1/2 protein expression in the hippocampus of mice in each group; **C** TUNEL staining was used to detect neuronal apoptosis in the hippocampus of mice in each group (×400); **D** BF-188 staining was used to detect changes in amyloid plaques and neurofibrillary tangles in the hippocampal region of mice in each group (×200, white arrows indicate amyloid plaques, red arrows indicate neurofibrillary tangles); n = 10, *P < 0.05 compared with blank group, ^#^P < 0.05 compared with HF group

Central insulin resistance is the core mechanism of cognitive impairment in T2DM (Arnold et al. 2018). Western blot results showed that compared with the blank group, the expression of brain hippocampal INSR, IRS-1, and IRS-2, as well as the insulin signaling-related pathways PI3K, p-AKT, Ras, and p-Erk1/2 proteins, were significantly decreased in mice in the HF group, while the expression of brain hippocampal INSR, IRS-1, and IRS-2, as well as the insulin signaling-related pathways PI3K, p-AKT, Ras, and p-Erk1/2 proteins, were significantly increased in mice in the HF + AE group (Fig. 2A, B). TUNEL staining results showed that compared with the blank group, the apoptosis of hippocampal neurons in mice in the HF group was significantly increased, while the apoptosis of hippocampal neurons in mice in the HF + AE group was significantly decreased (Fig. 2C). BF-188 staining results showed that compared with the blank group, the number of senile plaques and neurofibrillary tangles in the hippocampal region of the brain was significantly increased in the HF group, while those were significantly decreased in the HF + AE group (Fig. 2D).

In summary, the T2DM mouse model was successfully established and accompanied by cognitive impairment, while AE can alleviate cognitive impairment in T2DM mice.

### Up-regulation of MALAT1 expression in serum Exos of AE-treated T2DM mice

**Fig. 3:**
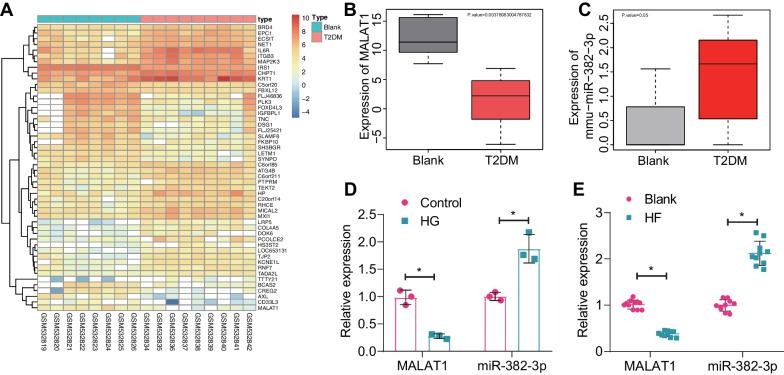
Screening of differentially expressed genes in serum-Exos from T2DM mice by transcriptome sequencing analysis. **A** The expression heat map of the top 50 differentially expressed genes with the smallest P. value, blue to red, represents increasing expression values; **B** the expression box plot of MALAT1 in serum-Exos from T2DM mice and blank mice (blank: n = 10, T2DM: n = 10); **C** the expression box plot of miR-382-3p in serum-Exos from T2DM mice and blank mice (blank: n = 10, T2DM: n = 10); **D** detection of MALAT1 and miR-382-3p expression in hippocampal neurons of T2DM mice by RT-qPCR (P < 0.05 compared with the control group); **E** Detection of MALAT1 and miR-382-3p expression levels in brain hippocampal tissue in mouse model by RT-qPCR (n = 10, *P < 0.05 compared with the blank group); n = 10, *P < 0.05 compared with the blank + Exos group, ^#^P < 0.05 compared with the HF + Exos group. All cell experiments were performed three times

Transcriptome sequencing analysis identified 1653 differentially expressed genes, from which we selected the top 50 based on the smallest P-values for generating the expression heatmap (Fig. 3A). Using the MNDR database, we obtained a list of 11 T2DM-associated LncRNAs (Additional file 3: Table S6), with MALAT1 receiving the highest score. Additionally, MALAT1 expression was significantly decreased in peripheral blood-derived exosomes from T2DM mice (Fig. 3B). Therefore, we postulate that MALAT1 is more closely related to T2DM.

Further screening of T2DM-associated miRNAs revealed a significant increase in the expression of miR-382-3p in T2DM (Fig. 3C). Diabetes can lead to hippocampal atrophy, neuronal loss, synapse loss, and damage (Jackson-Guilford et al. 2000; Trudeau et al. 2004). In the development of T2DM, some patients experience cognitive impairments and behavioral deficits characterized by neuronal damage and memory loss (Wang et al. 2020). In T2DM model mice, there is a significant reduction in the number of hippocampal neurons, and abnormal hippocampal neurons are observed under an optical microscope (Wang et al. 2016). Furthermore, studies have shown that neuroinflammation and oxidative stress may be key factors in the development of diabetes-associated cognitive impairments (Muriach et al. 2014; Wang et al. 2019). Therefore, in this study, neurons were selected to investigate the mechanisms related to disease impact through in vitro model. Primary neurons are more representative and have been used in similar previous studies (Akhtar et al. 2016, Li et al. 2023). Expression determination showed a significant decrease in MALAT1 expression and a significant increase in miR-382-3p expression in the HG group compared to the control group, while relative to the blank group, a significant decrease in MALAT1 expression and a significant increase in miR-382-3p expression were found in the HF group (Fig. 3D, E).

**Fig. 4:**
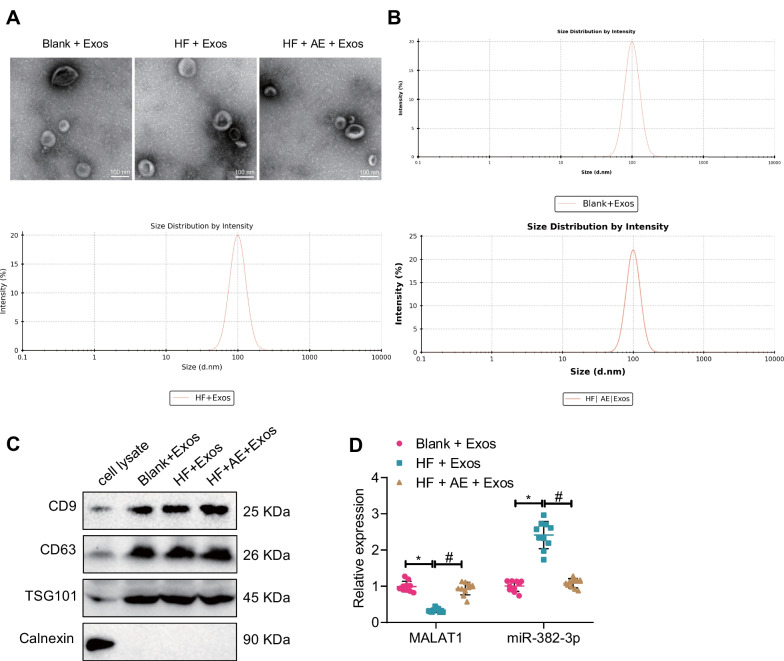
Changes in MALAT1 and miR-382-3p expression in serum-Exos from T2DM mice after AE. **A** Transmission electron microscope observation of Exos structure (100 nm); **B** dynamic light scattering detection of Exos size; **C** Western blot detection of CD9, CD63, TSG101, and Calnexin expression; **D** RT-qPCR detection of MALAT1 and miR-382-3p expression in mouse serum Exos of each group; n = 10, *P < 0.05 compared with the blank + Exos group, #, compared with the HF + Exos group. All cell experiments were performed three times

Following collection of serum and serum-Exos from T2DM mice, we found that the Exos had a saucer-like structure and a clear membrane structure (Fig. 4A). Based on the DLS, we estimated the size range of Exos to be 80–120 nm (Fig. 4B). In addition, western blot results showed that CD9, CD63, and TSG101 were higher in Exos, but the endoplasmic reticulum protein Calnexin expression was low (Fig. 4C). These results indicated that we successfully extracted Exos from the serum of T2DM mice.

Finally, we performed RT-qPCR to evaluate the expression of miR-382-3p and MALAT1 in serum Exos. The results showed that compared with the blank + Exos group, the expression of MALAT1 significantly decreased, but the expression of miR-382-3p significantly increased in the HF + Exos group. Conversely, compared with the HF + Exos group, the expression of MALAT1 significantly increased, and the expression of miR-382-3p significantly decreased in the HF + AE Exos group (Fig. 4D).

Collectively, MALAT1 and miR-382-3p are present in serum-Exos of T2DM mice, and AE can increase the expression of MALAT1 in mouse serum-Exos, thereby competitively inhibiting miR-382-3p expression.

### Serum-Exos can transmit MALAT1 and miR-382-3p into the brain parenchyma through the blood–brain barrier, choroid plexus epithelium, and CSF

We found that serum-Exos isolated from T2DM mice contained MALAT1. To further verify whether these Exos could pass through the blood–brain barrier and enter brain tissue, in vitro and in vivo experiments were conducted. In the in vitro experiment, it was observed under confocal fluorescence microscopy that there was a significant uptake of PKH67-labeled serum-Exos by neurons after co-culturing for 48 h (Additional file 1: Fig. S1A). RT-qPCR detection results showed that MALAT1 and miR-382-3p expression in the control + Exos group was significantly increased compared with the control + PBS group (normal neurons added with PBS) (Additional file 1: Fig. S1B).

In the in vivo experiment, after injecting PKH67-labeled serum-Exos into the lateral ventricle of HF mice for 5 h, RT-qPCR detection results showed that in the choroid plexus epithelium (CPE), CSF, and brain parenchyma in HF mice, MALAT1 and miR-382-3p expression in the Exos group was significantly increased compared with the PBS group, with MALAT1 expression being greater than that of miR-382-3p. Compared with the Exos + DMSO group, MALAT1 and miR-382-3p expression in the Exos + GW4869 group was significantly reduced in HF mice (Additional file 1: Fig. S1C–E).

Confocal fluorescence microscopy showed that Exos were expressed throughout the brain, including the hippocampus (Additional file 1: Fig. S1F). In addition, RT-qPCR detection of hippocampal tissue showed that MALAT1 and miR-382-3p expression in the Exos group was significantly increased compared with the PBS group, while in the Exos + GW4869 group, MALAT1 and miR-382-3p expression was significantly reduced (Additional file 1: Fig. S1G).

Therefore, the above results indicate that serum-Exos can enter the brain parenchyma through the blood–brain barrier, CPE, and CSF, thus transmitting MALAT1 and miR-382-3p.

### AE-mediated serum-Exos promotes hippocampal neuron proliferation and inhibits apoptosis and insulin resistance, improving cognitive impairment in T2DM mice

**Fig. 5:**
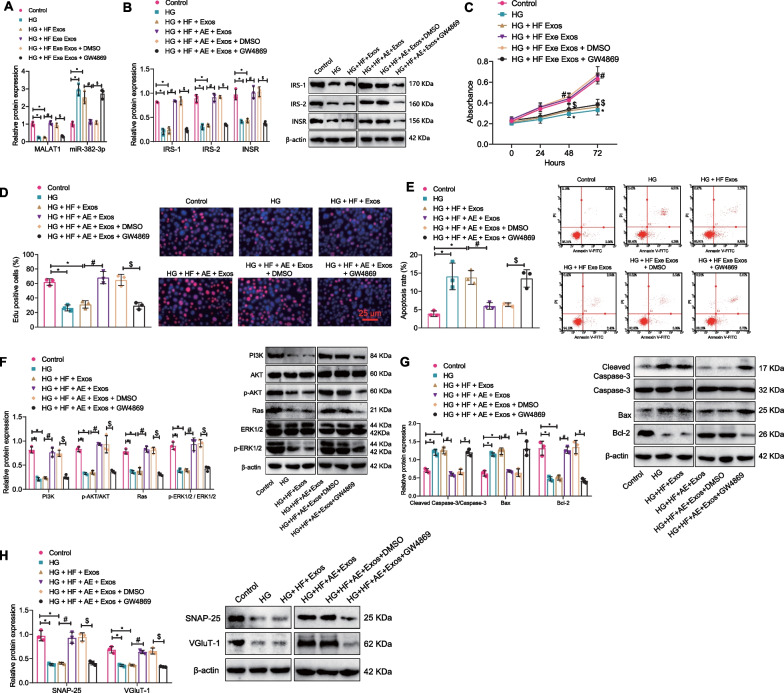
Effects of AE-mediated serum Exos on proliferation and apoptosis of hippocampal neurons. **A** RT-qPCR detection of MALAT1 and miR-382-3p expression in neurons; **B** Western blot detection of INSR, IRS-1, and IRS-2 expression in hippocampal neurons; **C** CCK-8 detection of hippocampal neuron activity in each group; **D** EdU experiment was used to detect hippocampal neuron apoptosis in each group; **E** Flow cytometry was used to detect hippocampal neuron apoptosis in each group; **F** Western blot detection of PI3K, AKT, p-AKT, Ras, Erk1/2, and p-Erk1/2 expression in hippocampal neurons; **G** Western blot detection of Cleaved Caspase-3, Caspase-3, Bax, and BCL-2 expression in hippocampal neurons; **H** Western blot detection of SNAP-25 and VGluT-1 expression in hippocampal neurons; *P < 0.05 compared with the control group, ^#^P < 0.05 compared with the HG + HF + Exos group, ^$^P < 0.05 compared with the HG + HF + AE + Exos + DMSO group; all cell experiments were repeated three times

Previous research has shown that serum-Exos has a neuroprotective effect in a mouse model of Parkinson's disease (Sun et al. 2020). Therefore, this study further investigated how AE mediates the effects of serum-Exos on neurons. RT-qPCR detection revealed that relative to the control group, decreased MALAT1 but increased miR-382-3p were found in the HG and HG + HF + Exos groups. Relative to the HG + HF + Exos group, increased MALAT1 but decreased miR-382-3p were seen in the HG + HF + AE + Exos group; relative to the HG + HF + AE + Exos + DMSO group, decreased MALAT1 but increased miR-382-3p were found in the HG + HF + AE + Exos + GW4869 group (Fig. 5A). Western blot results showed that the expression of INSR, IRS-1, and IRS-2 was decreased significantly in the HG and HG + HF + Exos groups compared to the control group. Compared with the HG + HF + Exos group, the expression of INSR, IRS-1, and IRS-2 was increased significantly in the HG + HF + AE + Exos group. Moreover, compared with the HG + HF + AE + Exos + DMSO group, the expression of INSR, IRS-1, and IRS-2 was decreased significantly in the HG + HF + AE + Exos + GW4869 group (Fig. 5B).

CCK-8, EdU staining, and flow cytometry experiments showed that compared with the control group, cell viability and proliferation in the HG and HG + HF + Exos groups decreased significantly while the apoptosis rate increased significantly. Compared with the HG + HF + Exos group, cell viability and proliferation in the HG + HF + AE + Exos group increased significantly, and the apoptosis rate decreased significantly. In addition, compared with the HG + HF + AE + Exos + DMSO group, cell viability and proliferation decreased significantly, and the apoptosis rate increased significantly in the HG + HF + AE + Exos + GW4869 group (Fig. 5C–E).

Western blot results also showed that compared with the control group, the expression of Cleaved Caspase-3 and Bax increased significantly, while the expression of PI3K, p-AKT, Ras, p-Erk1/2, BCL-2, SNAP-25, and VGluT-1 decreased significantly in the HG and HG + HF + Exos groups. Compared with the HG + HF + Exos group, the expression of PI3K, p-AKT, Ras, p-Erk1/2, BCL-2, SNAP-25, and VGluT-1 increased significantly, and the expression of Cleaved Caspase-3 and Bax decreased significantly in the HG + HF + AE + Exos group. In addition, compared with the HG + HF + AE + Exos + DMSO group, the expression of PI3K, p-AKT, Ras, p-Erk1/2, BCL-2, SNAP-25, and VGluT-1 decreased significantly, and the expression of Cleaved Caspase-3 and Bax increased significantly in the HG + HF + AE + Exos + GW4869 group (Fig. 5F–H).

**Fig. 6:**
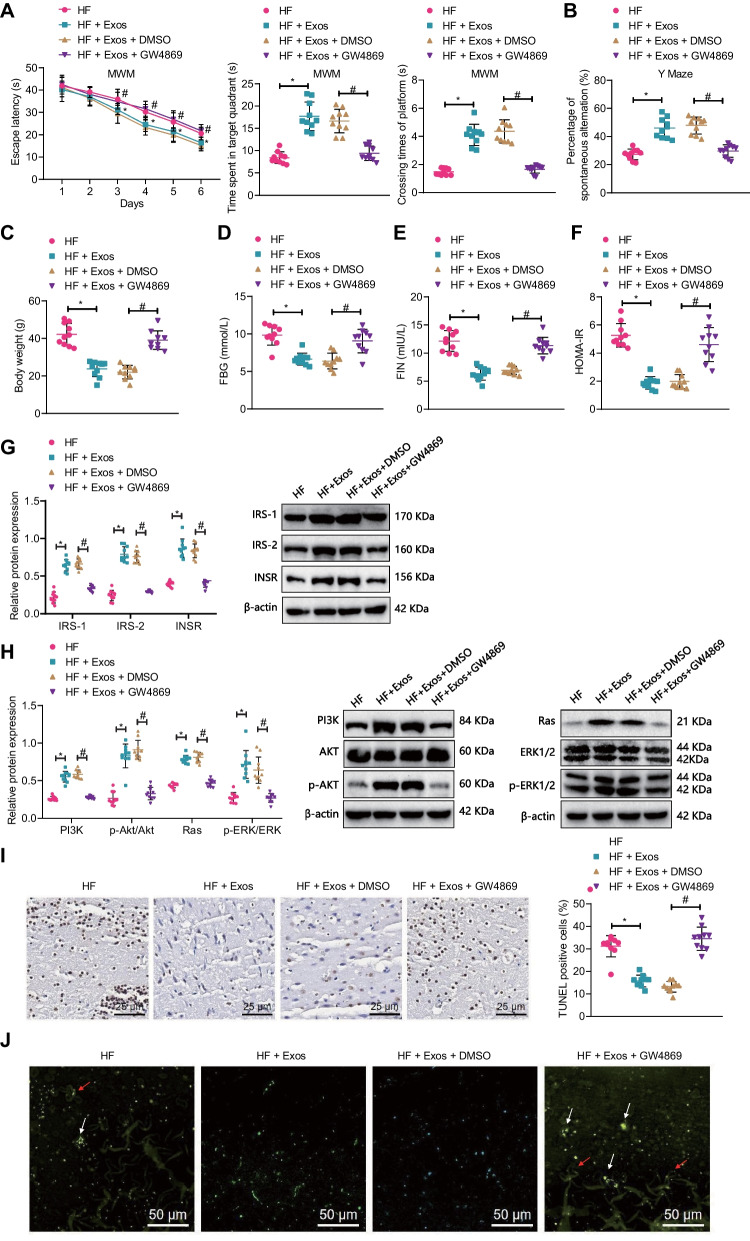
Effect of AE-mediated serum-Exos on cognitive impairment in T2DM mice. **A** Water maze test was used to detect the escape latency time during the platform searching experiment, time spent in the target quadrant, and the time spent crossing the target platform in each group of mice; **B** Y-maze test was used to detect the percentage of spontaneous alteration in each group of mice; **C** changes in body weight in each group of mice; **D** ELISA detection of fasting blood glucose levels in each group of mice; **E** ELISA detection of fasting serum insulin levels in each group of mice; **F** Comparison of insulin resistance index of each group of mice; **G** Western blot detection of INSR, IRS-1, and IRS-2 protein expression in mouse hippocampus; **H** Western blot detection of PI3K, AKT, p-AKT, Ras, Erk1/2, and p-Erk1/2 expression in mouse hippocampus; **I** TUNEL staining was used to detect the apoptosis of brain hippocampal neurons in each group of mice (×400); **J** BF-188 staining was used to detect changes in senile plaques and neurofibrillary tangles in the brain hippocampus of each group of mice (×200, white arrows indicate senile plaques, red arrows indicate neurofibrillary tangles); n = 10, *P < 0.05 compared with the HF group, ^#^P < 0.05 compared with the HF + Exos + DMSO group

Furthermore, we validated the use of HF + AE + Exos (Exos) in a mouse model of T2DM. The water maze and Y maze experiments showed that compared with the HF group, the mice in the HF + Exos group had a significantly reduced the escape latency time during the platform searching experiment and increased the time spent in target quadrant, platform crossings, and spontaneous alternation percentage. Compared with the HF + Exos + DMSO group, the escape latency time during the platform searching experiment was increased, but the time spent in target quadrant, platform crossings, and spontaneous alternation percentage was decreased in the HF + Exos + GW4869 group (Fig. 6A, B).

Additionally, compared with the HF group, the mice in the HF + Exos group had significantly decreased weight, FBG, FIN, and HOMA-IR. Compared with the HF + Exos + DMSO group, the mice in the HF + Exos + GW4869 group had significantly increased weight, FBG, FIN, and HOMA-IR (Fig. 6C–F). Western blot results showed that compared with the HF group, the expression of INSR, IRS-1, IRS-2, PI3K, p-AKT, Ras, and p-Erk1/2 significantly increased in the hippocampus of mice in the HF + Exos group. Conversely, compared with the HF + Exos + DMSO group, the expression of INSR, IRS-1, IRS-2, PI3K, p-AKT, Ras, and p-Erk1/2 decreased significantly in the hippocampus of mice in the HF + Exos + GW4869 group (Fig. 6G, H).

Finally, TUNEL staining showed that the apoptosis of hippocampal neurons in the HF + Exos group decreased significantly compared with the HF group. Compared with the HF + Exos + DMSO group, the apoptosis of hippocampal neurons in the HF + Exos + GW4869 group increased significantly (Fig. 6I). BF-188 staining showed that compared with the HF group, the number of senile plaques and neurofibrillary tangles in the hippocampal region of mice in the HF + Exos group decreased significantly. Compared with the HF + Exos + DMSO group, the number of senile plaques and neurofibrillary tangles in the hippocampal region of mice in the HF + Exos + GW4869 group increased significantly (Fig. 6J).

In summary, AE can inhibit high glucose-induced hippocampal neuron apoptosis and insulin resistance and promote neuron proliferation through mediating serum-Exos thus improving cognitive impairment in T2DM mice.

### Silencing MALAT1 inhibits the insulin resistance and neuroprotection of serum-Exos mediated by AE in T2DM mice

**Fig. 7:**
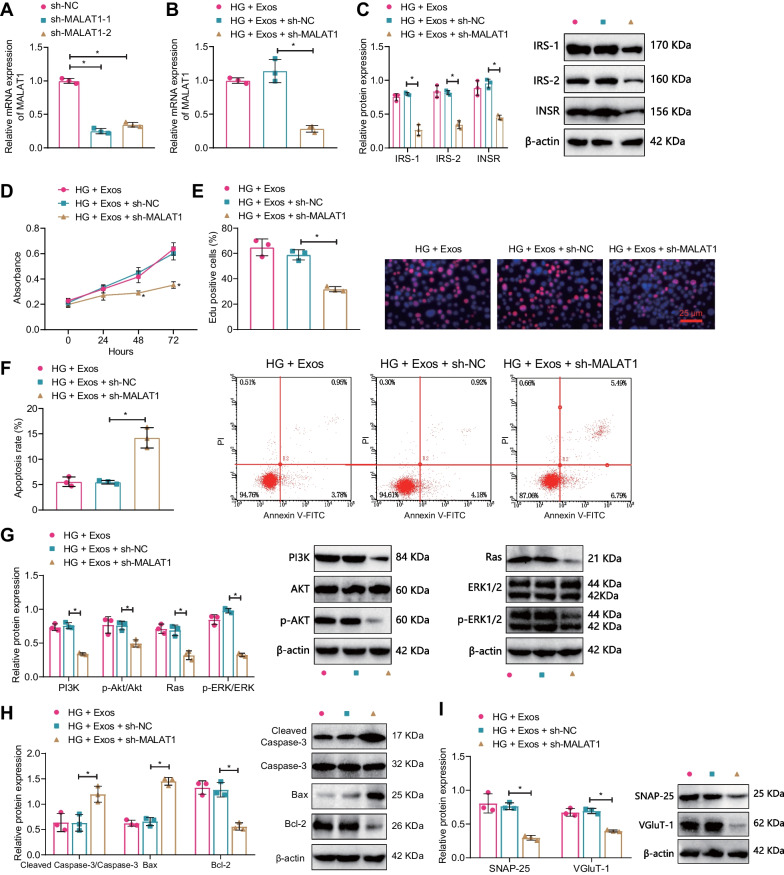
Effects of silent MALAT1 on hippocampal neuron apoptosis. **A** RT-qPCR detection of the efficiency of MALAT1 silencing (*P < 0.05 compared with the sh-NC group); **B** RT-qPCR detection of MALAT1 expression in hippocampal neurons of each group; **C** Western blot detection of INSR expression in hippocampal neurons; **D** CCK-8 detection of hippocampal neuron activity in each group; **E** EdU experiment was used to detect apoptosis of hippocampal neurons in each group; **F** flow cytometry was used to detect apoptosis of hippocampal neurons in each group; **G**–**I** Western blot detection of PI3K, AKT, p-AKT, Ras, Erk1/2, p- Erk1/2 expression; **H** Western blot detection of Cleaved Caspase-3, Caspase-3, Bax, BCL-2 expression; **I** Western blot detection of SNAP-25, VGluT-1 expression in hippocampal neurons; *P < 0.05 compared with the HG + Exos + sh-NC group; all cell experiments were repeated three times

Previous study has shown that running exercise can improve cognitive ability in mice with ischemia–reperfusion brain injury by upregulating MALAT1 expression and inhibiting hippocampal neuron cell apoptosis (Shang et al. 2018). Therefore, we constructed two MALAT1-silencing sequences, and RT-qPCR results showed that compared with the sh-NC group, the MALAT1 expression in the sh-MALAT1-1 and sh-MALAT1-2 groups was significantly reduced (Fig. 7A), and the efficiency of the sh-MALAT1-1 group was higher, so we used the sh-MALAT1-1 (sh-MALAT1) silencing sequence for subsequent experiments.

Further in vitro cell experiments were conducted for validation. RT-qPCR results showed that compared with the HG + Exos + sh-NC group, the MALAT1 expression in the HG + Exos + sh-MALAT1 group was significantly reduced; there was no significant difference between the HG + Exos and HG + Exos + sh-NC groups (Fig. 7B). Western blot results showed that compared with the HG + Exos + sh-NC group, the expression of INSR, IRS-1, and IRS-2 in the HG + Exos + sh-MALAT1 group was significantly reduced; there was no significant difference between the HG + Exos and HG + Exos + sh-NC groups (Fig. 7C). Results of CCK-8, EdU staining, and flow cytometry showed that compared with the HG + Exos + sh-NC group, the cell viability and proliferation of the HG + Exos + sh-MALAT1 group were significantly reduced, and the cell apoptosis rate was significantly increased; there was no significant difference between the HG + Exos and HG + Exos + sh-NC groups (Fig. 7D–F).

Western blot results showed that compared with the HG + Exos + sh-NC group, the expression of PI3K, p-AKT, Ras, p-Erk1/2, BCL-2, SNAP-25, and VGluT-1 was significantly reduced, but the expression of Cleaved Caspase-3 and Bax was significantly increased in the HG + Exos + sh-MALAT1 group (Fig. 7G–I).

**Fig. 8:**
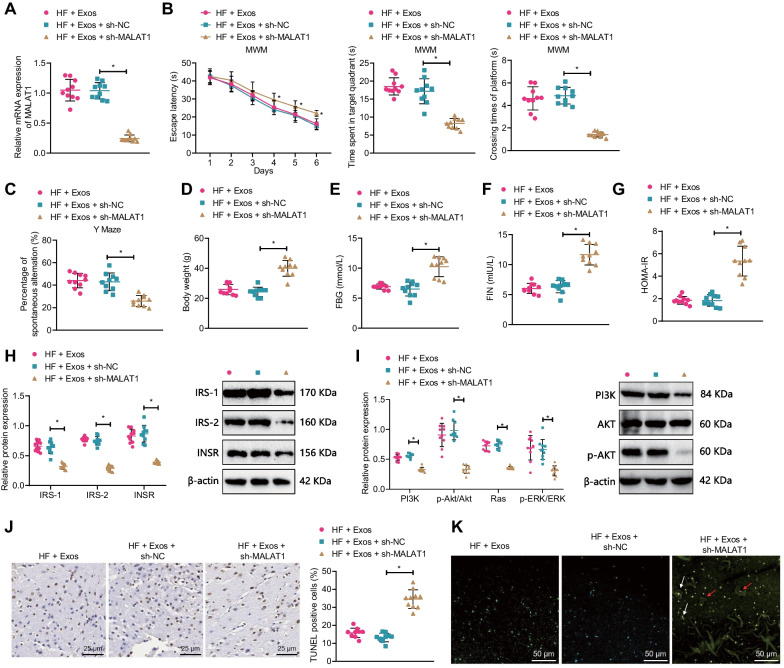
The effect of silencing MALAT1 on cognitive impairment in T2DM mice. **A** RT-qPCR detection of hippocampal MALAT1 expression in each group of mice; **B** Water maze test was used to detect the escape latency during the platform searching experiment, time spent in the goal quadrant, and time to cross the platform in each group of mice; **C** Y-maze test was used to detect spontaneous alternation percentage in each group of mice; **D** Changes in body weight of each group of mice; **E** ELISA detection of fasting blood glucose levels in each group of mice; **F** ELISA detection of fasting serum insulin levels in each group of mice; **G** Comparison of insulin resistance index in each group of mice; **H** Western blot detection of INSR, IRS-1, and IRS-2 expression in the hippocampus of each group of mice; **I** Western blot detection of PI3K, AKT, p-AKT, Ras, Erk1/2, and p-Erk1/2 expression in the hippocampus of each group of mice; **J** TUNEL staining was used to detect hippocampal neuronal apoptosis in each group of mice (×400); **K** BF-188 staining was used to detect changes in senile plaques and neurofibrillary tangling in the hippocampus of each group of mice (×200, white arrows indicate senile plaques, red arrows indicate neurofibrillary tangling); n = 10, * indicates P < 0.05 compared to the HF + Exos + sh-NC group

In animal experiments, RT-qPCR results showed that compared with the HF + Exos + sh-NC group, the MALAT1 expression in the HF + Exos + sh-MALAT1 group was significantly reduced; no significant difference was found between the HF + Exos and HF + Exos + sh-NC groups (Fig. 8A).

Results of the water maze and Y maze experiments showed that compared with the HF + Exos + sh-NC group, the escape latency time during the platform searching experiment was increased, but the time spent in target quadrant, platform crossings, and spontaneous alternation percentage decreased significantly in the HF + Exos + sh-MALAT1 group (Fig. 8B, C). In addition, compared with the HF + Exos + sh-NC group, the body weight, FBG, FIN, and HOMA-IR of mice in the HF + Exos + sh-MALAT1 group were all significantly increased (Fig. 8D–G).

Western blot results showed that compared with the HF + Exos + sh-NC group, the expression of INSR, IRS-1, IRS-2, and PI3K, p-AKT, Ras, and p-Erk1/2 in the hippocampus of mice in the HF + Exos + sh-MALAT1 group was significantly reduced (Fig. 8H, I). TUNEL staining and BF-188 staining showed that compared with the HF + Exos + sh-NC group, the apoptosis of hippocampal neurons in the brain of mice and the senile plaques and neurofibrillary tangles in the hippocampal area in the HF + Exos + sh-MALAT1 group were significantly increased (Fig. 8J–K).

The above results indicate that silencing MALAT1 can inhibit the protective effect of AE-mediated serum Exos on insulin resistance and cognitive impairment in T2DM mice.

### Overexpression of miR-382-3p can inhibit the insulin resistance and neuroprotective effects of serum-Exos mediated by AE in T2DM mice

**Fig. 9:**
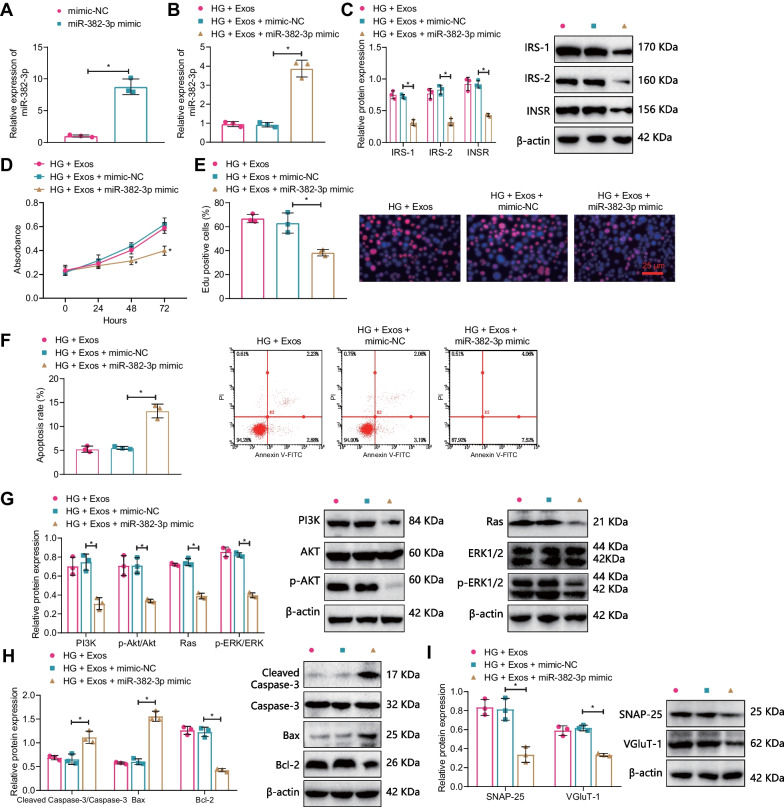
The effect of overexpressing miR-382-3p on hippocampal neuronal apoptosis. **A** RT-qPCR detection of overexpression efficiency of miR-382-3p (P < 0.05 compared with the mimic-NC group); **B** RT-qPCR detection of hippocampal neuronal miR-382-3p expression in each group; **C** Western blot detection of INSR, IRS-1, and IRS-2 expression in hippocampal neurons; **D** CCK-8 detection of hippocampal neuron activity in each group; **E** EdU experiment was used to detect hippocampal neuronal apoptosis in each group; **F** flow cytometry was used to detect hippocampal neuronal apoptosis in each group; **G** Western blot detection of PI3K, AKT, p-AKT, Ras, Erk1/2, and p-Erk1/2 expression in hippocampal neurons; **H** Western blot detection of Cleaved Caspase-3, Caspase-3, Bax, and BCL-2 expression levels in hippocampal neurons; **I** Western blot detection of SNAP-25 and VGluT-1 expression in hippocampal neurons; *P < 0.05 compared with the HG + Exos + mimic-NC group; n = 10, *P < 0.05 compared with the HF + Exos + agomir-NC group; all cell experiments were repeated three times

Previous studies have shown that miR-382-3p is highly expressed in the hippocampus of CUMS mice models and may inhibit hippocampal neuron function, while MALAT1 can target and inhibit the expression of miR-382-3p (Zhou et al. 2018a, b; Liu et al. 2019). To validate this conclusion, data on the overexpression of miR-382-3p were obtained through RT-qPCR. The results showed that miR-382-3p expression in the miR-382-3p mimic group significantly increased compared with the mimic-NC group (Fig. 9A).

In in vitro experiments, RT-qPCR detection results showed that compared with the HG + Exos + mimic-NC group, miR-382-3p expression significantly increased in the HG + Exos + miR-382-3p mimic group, while no significant difference was observed between the HG + Exos and HG + Exos + mimic-NC groups (Fig. 9B). Western blot results showed that compared with the HG + Exos + mimic-NC group, the expression of INSR, IRS-1, and IRS-2 significantly decreased in the HG + Exos + miR-382-3p mimic group. In contrast, there was no significant difference between the HG + Exos and HG + Exos + sh-NC groups (Fig. 9C). The results of CCK-8, EdU staining, and flow cytometry further indicated that compared with the HG + Exos + mimic-NC group, cell viability and proliferation significantly decreased, while the cell apoptosis rate significantly increased in the HG + Exos + miR-382-3p mimic group (Fig. 9D–F).

Western blot results showed that compared with the HF + Exos + mimic-NC group, the protein expressions of PI3K, p-AKT, Ras, p-Erk1/2, BCL-2, SNAP-25, and VGluT-1 were significantly decreased, while the expressions of Cleaved Caspase-3 and Bax increased in the HF + Exos + miR-382-3p mimic group (Fig. 9G–I).

**Fig. 10:**
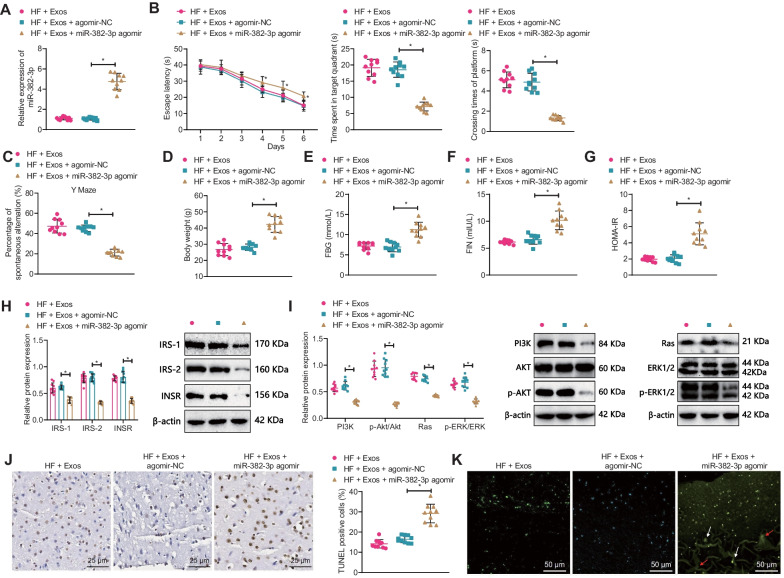
The effect of overexpressing miR-382-3p on hippocampal neuronal apoptosis and cognitive impairment in T2DM mice. **A** RT-qPCR detection of hippocampal miR-382-3p expression levels in each group of mice; **B** Water maze test was used to detect the escape latency during the platform searching experiment, time spent in the goal quadrant, and time to cross the platform in each group of mice; **C** Y-maze test was used to detect spontaneous alternation percentage in each group of mice; **D** changes in body weight of each group of mice; **E** ELISA detection of fasting blood glucose levels in each group of mice; **F** ELISA detection of fasting serum insulin levels in each group of mice; **G** Comparison of insulin resistance index in each group of mice; **H**–**I** Western blot detection of INSR, IRS-1, IRS-2, PI3K, AKT, p-AKT, Ras, Erk1/2, and p-Erk1/2 protein expression levels in each group of mice; J TUNEL staining was used to detect hippocampal neuronal apoptosis in each group of mice (×400); **K** BF-188 staining was used to detect changes in senile plaques and neurofibrillary tangling in the hippocampus of each group of mice (×200, white arrows indicate senile plaques, and red arrows indicate neurofibrillary tangling); n = 10, *P < 0.05 compared with the HF + Exos + agomir-NC group

In in vivo animal experiments, RT-qPCR detection showed that compared with the HF + Exos + agomir-NC group, miR-382-3p expression was increased in the HF + Exos + miR-382-3p agomir group, while no significant difference was observed between the HF + Exos and HF + Exos + agomir-NC groups (Fig. 10A). The results of the water maze and Y maze experiments further indicated that compared with the HF + Exos + agomir-NC group, the escape latency time during the platform searching experiment was increased, but the time spent in target quadrant, platform crossings, and spontaneous alternation percentage decreased significantly in the HF + Exos + miR-382-3p agomir group (Fig. 10B, C). Additionally, the body weight, FBG, FIN, and HOMA-IR of mice in the HF + Exos + miR-382-3p agomir group significantly increased compared with those in the HF + Exos + agomir-NC group (Fig. 10D–G).

Western blot results showed that compared with the HF + Exos + agomir-NC group, expressions of INSR, IRS-1, and IRS-2, as well as PI3K, p-AKT, Ras, and p-Erk1/2 in the mouse hippocampus significantly decreased in the HF + Exos + miR-382-3p agomir group (Fig. 10H, I). Additionally, TUNEL staining and BF-188 staining showed that compared with the HF + Exos + agomir-NC group, apoptosis significantly increased and the number of senile plaques, and neurofibrillary tangles in the hippocampal region of mice in the HF + Exos + miR-382-3p agomir group significantly increased (Fig. 10J, K).

In conclusion, overexpression of miR-382-3p can inhibit the protective effects of AE-mediated serum-Exos on insulin resistance and cognitive impairment in T2DM mice.

### Serum-Exosomes can transmit MALAT1 to competitively inhibit miR-382-3p and up-regulate BDNF expression in hippocampal neurons

Previous studies have shown that MALAT1 can inhibit apoptosis by upregulating BDNF expression through suppression of miR-382-3p in the central neurons of cerebral palsy mice. In addition, AE can promote hippocampal neuron regeneration and improve mouse memory and cognitive function by upregulating BDNF expression (Choi et al. 2018; Li et al. 2019; Liu et al. 2019).

**Fig. 11:**
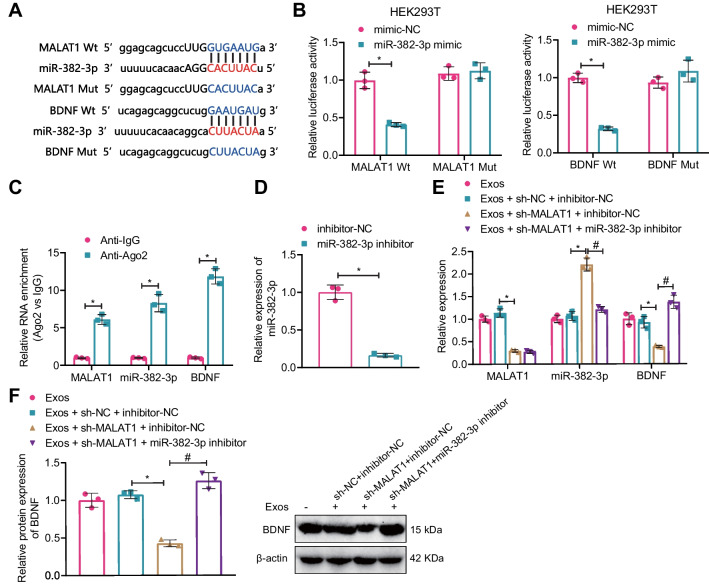
Verification of the mechanism of serum Exos-mediated regulation of miR-382-3p and BDNF expression through MALAT1 in hippocampal neurons. **A** StarBase and Targetscan online prediction websites predicted targeting binding sites of MALAT1 with miR-382-3p and miR-382-3p with BDNF; **B** dual-luciferase experiments verified the targeting relationships between MALAT1 and miR-382-3p, and miR-382-3p and BDNF (P < 0.05 compared with the mimic-NC group); **C** RIP experiments verified the enrichment levels of MALAT1, miR-382-3p, and BDNF binding with AGO2 (P < 0.05 compared with the Anti-lgG group); **D** RT-qPCR detection of miR-382-3p inhibition efficiency (P < 0.05 compared with the inhibitor-NC group); **E** RT-qPCR detection of MALAT1 and miR-382-3p expression in each group of neurons (*P < 0.05 compared with the Exos + sh-NC + inhibitor-NC group, ^#^P < 0.05 compared with the Exos + sh-MALAT1 + inhibitor-NC group); **F** Western blot detection of BDNF expression levels in each group of neurons; *P < 0.05 compared with the Exos + sh-NC + inhibitor-NC group, ^#^P < 0.05 compared with the Exos + sh-MALAT1 + inhibitor-NC group; all cell experiments were repeated three times

Prediction by the online prediction websites StarBase and Targetscan showed that there were targeting binding sites between MALAT1 and miR-382-3p, as well as between miR-382-3p and BDNF (Fig. 11A). Dual-luciferase reporter gene experiments suggested that the luciferase activity significantly decreased in the co-transfection groups of miR-382-3p mimic with MALAT1 Wt, or miR-382-3p mimic with BDNF Wt compared with the mimic-NC group, while there was no significant difference in the MALAT1 Mut or BDNF Mut groups (Fig. 11B). RIP assay found that the expression of MALAT1, miR-382-3p, and BDNF was significantly increased in the Anti-Ago2 group compared with the Anti-lgG group (Fig. 11C).

RT-qPCR detection showed that the expression of miR-382-3p was significantly decreased in the miR-382-3p inhibitor group compared with the inhibitor-NC group (Fig. 11D). Additionally, the expression of MALAT1 and BDNF was significantly decreased while the expression of miR-382-3p was significantly increased in the Exos + sh-MALAT1 + inhibitor-NC group compared with the Exos + sh-NC + inhibitor-NC group. Compared with the Exos + sh-MALAT1 + inhibitor-NC group, the expression of miR-382-3p was significantly decreased, while the expression of BDNF was significantly increased in the Exos + sh-MALAT1 + miR-382-3p inhibitor group (Fig. 11E, F).

These results indicate that serum-exosomes can transmit MALAT1 to competitively inhibit miR-382-3p and up-regulate BDNF expression in hippocampal neurons.

## Discussion

It has been widely proved that insulin resistance is a determining factor in the pathophysiology of T2DM, and exercise has been demonstrated to be capable of improving insulin resistance (Sampath Kumar et al. 2019). Notably, in recent years, nucleic acids containing exosomes-especially miRNAs and lncRNAs-have been revealed to modulate communications between organs in pathological processes of diabetes, including affecting metabolic signals and insulin signals in target tissues and cell viability (Chang and Wang 2019; Marttila et al. 2021). Here, this study revealed the important role of non-coding RNA in the pathogenesis of diabetes-related cognitive impairment. In this study, we investigated the roles of MALAT1 and miR-382-3p in T2DM-related cognitive impairment and the therapeutic potential of AE in improving cognitive function in T2DM mice. The MALAT1/miR-382-3p/BDNF signaling pathway may play a key regulatory role in cognitive impairment in T2DM. It provides a new research direction for further exploration of the pathophysiological mechanisms of diabetes-related cognitive impairment.

Following modeling establishment, we identified that AE may alleviate cognitive impairment in T2DM mice, suggesting that it may serve as a potential therapeutic approach to treating cognitive impairment in T2DM patients. It has been reported that T2DM is intensively correlated with lower performance on multiple domains of cognitive function and with structural abnormalities of the brain (Damanik and Yunir 2021). Physical activity is widely advocated in the management of T2DM, and moreover, a mounting body of evidence suggests that exercise, which is a specific type of physical activity, has significant positive effects on the maintenance and enhancement of brain structure as well as function (Callisaya and Nosaka 2017). Similarly, AE has been recently confirmed as one effective way to improve T2DM-related cognitive impairment (Lin et al. 2022).

We also found elevated MALAT1 expression in serum-Exos in T2DM after AE. Specifically, AE can increase the expression of MALAT1 in serum-exosomes, competitively inhibit miR-382-3p, and upregulate BDNF expression, leading to improvements in cognitive function in T2DM mice. Exosomes have become an important carrier for intercellular communication, and their role in T2DM and AE has also been studied (Chang and Wang 2019; Estebanez et al. 2021). A previous study has demonstrated the role of exosomes in mediating intercellular communication and the importance of delivering ncRNAs to target cells for therapy (Kalluri and LeBleu 2020). In addition, the involvement of exosomes in regulating glucose metabolism and insulin sensitivity, which are critical physiological processes in T2DM, has also been documented (Gauthier et al. 2022; Xu et al. 2022). Importantly, the critical role of exosomal lncRNAs has been widely documented in diabetes mellitus (Gauthier et al. 2022). MALAT1 is a long non-coding RNA that participates in various cellular processes, including alternative splicing and gene regulation (Matuszyk 2022). Previous studies have shown that MALAT1 is upregulated in the hippocampus of diabetic rats, resulting in cognitive impairment (Du et al. 2020). Consistently, a decreased MALAT1 level was also detected in serum-derived EVs from T2DM individuals when compared to controls (Tello-Flores et al. 2020). miRNAs, specialized short non-coding RNAs (20–22 nt), have been highlighted for their usefulness as biomarkers of diseases (Vasu et al. 2019). Moreover, miR-382 paired with miR-370 has been confirmed as a sensitive biomarker for the detection of mild cognitive impairment (Salama et al. 2020). Furthermore, our findings are consistent with previous research that found AE improved cognitive function in diabetic rats and increased expression of BDNF (Jesmin et al. 2022). In line with our findings, a previous study also unveiled that decreased MALAT1 expression caused by exercise could suppress insulin resistance in T2DM via increasing miR-382-3p expression through synchronous inhibition of resistin (Liu et al. 2019).

## Conclusion

In summary, we can tentatively conclude that AE may improve cognitive impairment in T2DM mice by upregulating MALAT1 expression in serum-exosomes, competitively inhibiting miR-382-3p, and upregulating BDNF expression, thereby inhibiting hippocampal neuron apoptosis (Additional file 2: Fig. S2). This study demonstrates that AE has a therapeutic effect on improving cognitive impairment in diabetes. Furthermore, our in vivo experiments have confirmed that AE-mediated treatment significantly improves cognitive impairment in T2DM mice. It provides new insights into treating diabetes-associated cognitive impairment, which may promote personalized and differentiated diabetes treatment.

Although this study explores the role of AE and Exos in diabetes-associated cognitive impairment, there are still some shortcomings. First, the T2DM mouse model used may not fully simulate the pathological status of human diabetes, and some experimental results need further clinical validation. In addition, the results of this study have not been directly validated against human diabetes-associated cognitive impairment, and further research is needed to verify its clinical application prospects. Moreover, this study focuses on the regulatory role of the MALAT1/miR-382-3p/BDNF signaling pathway; other signaling pathways may also have an impact. Thus, future research should combine more molecular biology and cell biology techniques to further elucidate the role of the MALAT1/miR-382-3p/BDNF signaling pathway in diabetes-associated cognitive impairment.

### Supplementary Information

**Additional file 1: Fig. S1.** Experimental verification of whether serum Exos can pass through the blood–brain barrier. Note: (A) Observation of neuron uptake of Exos by confocal fluorescence microscopy (400 ×); (B) RT-qPCR detection of MALAT1 and miR-382-3p expression in hippocampal neurons (P < 0.05 compared with the control + PBS group); C–E: RT-qPCR detection of MALAT1 and miR-382-3p expression in CPE, CSF, and brain parenchyma; (F) Observation of brain and hippocampal uptake of Exos by confocal fluorescence microscopy (20 × , 100 ×); (G) RT-qPCR detection of MALAT1 and miR-382-3p expression in mouse brain hippocampal tissue; n = 10, *, P < 0.05 compared with the PBS group, #, P < 0.05 compared with the Exos + DMSO group; all cell experiments were repeated three times.

**Additional file 2: Fig. S2.** Schematic diagram illustrating the molecular mechanism of aerobic exercise improving cognitive impairment in T2DM mice by regulating the MALAT1/miR-382-3p/BDNF signaling pathway in serum-exosomes.

**Additional file 3: Table S1.** In vivo experimental grouping and treatments. **Table S2. **shRNA sequences. **Table S3.** In vitro cell experimental grouping and treatments. **Table S4. **The primer sequence of RT-qPCR. **Table S5.** Primary antibody product information. **Table S6. **T2DM-related lncRNAs obtained from the MNDR database.

## Data Availability

The data that supports the findings of this study are available on request from the corresponding author.
