# Supplementary material for: Aerobic exercise improves cognitive impairment in mice with type 2 diabetes by regulating the MALAT1/miR-382-3p/BDNF signaling pathway in serum-exosomes
Source: Mol Med. 2023 Sep 22;29:130. doi: 10.1186/s10020-023-00727-1 (PMC10517522; doi:10.1186/s10020-023-00727-1)
Supplement: Supplementary file 3 — Additional file 3: Table S1. In vivo experimental grouping and treatments. Table S2. shRNA sequences. Table S3. In vitro cell experimental grouping and treatments. Table S4. The primer sequence of RT-qPCR. Table S5. Primary antibody product information. Table S6. T2DM-related lncRNAs obtained from the MNDR database. [file 10020_2023_727_MOESM3_ESM.docx]

**Table S1** In vivo experimental grouping and treatments

| Group | Treatment |
| --- | --- |
| Blank | Normal-diet control group |
| HF | High fat diet group |
| HF+AE | Exercise training group of mice fed on a high-fat diet |
| HF+Exos | High-fat diet mice were injected with the AE-Exos group |
| HF+Exos+DMSO | Mice on a high-fat diet were injected with the AE-Exos and the DMSO group |
| HF+Exos+GW4869 | Mice on a high-fat diet were injected with the AE-Exos and the GW4869 group |
| HF+Exos+sh-NC | The high-fat diet mice were injected with AE-Exos and silenced lentiviral controls |
| HF+Exos+sh-MALAT1 | High-fat diet mice were injected with AE-Exos and the silent MALAT1 lentivirus group |
| HF+Exos+agomir-NC | Mice on a high-fat diet were injected with AE-Exos and miRNA agonist controls |
| HF+Exos+miR-382-3p agomir | High-fat diet mice were injected with AE-Exos and miR-382-3p agonist groups |

Note: HF, High-fat ; Exos, Extracellular vesicles ; DMSO, Dimethyl sulfoxide ; AE, Androgen receptor ; sh-NC, Non-targeting short hairpin RNA ; MALAT1, Metastasis-associated lung adenocarcinoma transcript 1 ; agomir-NC, Non-targeting miRNA agomir.

**Table S2** shRNA sequences

| shRNA | Sequence (5’-3’) |
| --- | --- |
| sh-NC | CCTAAGGTTAAGTCGCCCTCG |
| sh-MALAT1 (sh-MALAT1-1) | GGAAGTGAAAGACGAAGAAGA |
| sh-MALAT1-2 | GAAGAAGACATACAGGAAGGT |

**Table S3** In vitro cell experimental grouping and treatments

| Group | Treatment |
| --- | --- |
| Control | Control group with normal culture of neurons |
| HG | High glucose culture: medium in 45 mmol L / L glucose for 48 h |
| HG+HF+Exos | High-glucose culture, Exo treatment of mice on a high-fat diet |
| HG+HF+AE+Exos | High-glucose culture, high-fat diet and exercise trained mice treated with Exo |
| HG+HF+AE+Exos+DMSO | High-glucose culture, high-fat diet and exercise training mice for solvent DMSO treatment of Exo and GW4869 |
| HG+HF+AE+Exos+GW4869 | High-glucose cultures, high-fat diet and exercise trained mice treated with Exo and GW4869 |
| sh-NC | Silenced by the lentiviral control group |
| sh-MALAT1-1 | Silencing of the MALAT1 lentiviral group 1 |
| sh-MALAT1-2 | Silencing of MALAT1 in the lentiviral group 2 |
| HG+Exos+sh-NC | High-glucose culture, high-fat diet and exercise training mice with Exo, silent lentiviral control treatment |
| HG+Exos+sh-MALAT1 | High-glucose culture, high-fat diet and exercise training mice with Exo, silenced MALAT1 lentiviral treatment |
| mimic-NC | And miR-382-3p mimic control treatment |
| miR-382-3p mimic | With miR-382-3p mimic treatment |
| HG+Exos+mimic-NC | High glucose culture, mice with Exo, miR-382-3p mimic control with high fat diet and exercise training |
| HG+Exos+miR-382-3p mimic | High-glucose culture, high-fat diet and exercise training mice of Exo, miR-382-3p mimic treatment |
| inhibitor-NC | And miR-382-3p inhibitor control treatment |
| miR-382-3p inhibitor | With miR-382-3p inhibitor treatment |
| Exos+sh-NC+inhibitor-NC | High-glucose culture, high-fat diet and exercise training mice with Exo, silent lentiviral control and miR-382-3p inhibitor control treatment |
| Exos+sh-MALAT1+inhibitor-NC | High-glucose culture, Exo, silent MALAT1 lentivirus treatment of high-fat diet and exercise training mice, and miR-382-3p inhibitor control treatment |
| Exos+sh-MALAT1+miR-382-3p inhibitor | High-glucose culture, high-fat diet and exercise-trained mice with Exo, silent-MALAT1 lentiviral treatment, and miR-382-3p inhibitor treatment |

Note: HF, High-fat; Exos, exosomes; DMSO, Dimethyl sulfoxide ; AE, Androgen receptor ; sh-NC, Non-targeting short hairpin RNA ; MALAT1, Metastasis-associated lung adenocarcinoma transcript 1.

**Table S4** The primer sequence of RT-qPCR

| Target | Sequence (5’-3’) |
| --- | --- |
| MALAT1 (human) | Forward: AAAGTCCGCCATTTTGCCAC  Reverse: CTCACAAAACCCCCGGAACT |
| MALAT1 (mouse) | Forward: TGCAGTGTGCCAATGTTTCG  Reverse: GGCCAGCTGCAAACATTCAA |
| miR-382-3p (human) | Forward: AATCATTCACGGACAACACTT  Reverse: Reverse universal primer |
| miR-382-3p (mouse) | Forward: TCATTCACGGACAACACTTTTT  Reverse: Reverse universal primer |
| β-actin (human) | Forward: CTTCGCGGGCGACGAT  Reverse: CCACATAGGAATCCTTCTGACC |
| β-actin (mouse) | Forward: TGAGCTGCGTTTTACACCCT  Reverse: GCCTTCACCGTTCCAGTTTT |
| U6 (human, mouse) | Forward: CTCGCTTCGGCAGCACA  Reverse: Reverse universal primer |

**Table S5** Primary antibody product information

| Antibody Name | Dilution Ratio | Catalog Number | Manufacturer |
| --- | --- | --- | --- |
| β-actin | 1: 5000 | 4970 | Cell Signaling Technology, USA |
| BDNF | 1: 1000 | ab108319 | Abcam, UK |
| INSR | 1: 1000 | ABK1-A6730 | Abyntek |
| IRS-1 | 1: 1000 | ab52167 | Abcam, UK |
| IRS-2 | 1: 1000 | ab134101 | Abcam, UK |
| PI3K | 1: 1000 | ab278545 | Abcam, UK |
| AKT | 1: 1000 | ab8805 | Abcam, UK |
| p-AKT | 1: 1000 | 4060 | Cell Signaling Technology, USA |
| Ras | 1: 1000 | 67648 | Cell Signaling Technology, USA |
| p-Erk1/2 | 1: 1000 | 4370 | Cell Signaling Technology, USA |
| Erk1/2 | 1: 1000 | 4695 | Cell Signaling Technology, USA |
| Caspase-3 | 1: 1000 | ab184787 | Abcam, UK |
| Cleaved Caspase-3 | 1: 1000 | 9661 | Cell Signaling Technology, USA |
| Bax | 1: 1000 | ab32503 | Abcam, UK |
| BCL-2 | 1: 1000 | ab182858 | Abcam, UK |
| SNAP25 | 1: 1000 | 5309 | Cell Signaling Technology, USA |
| VGluT-1 | 1: 1000 | ab227805 | Abcam, UK |
| CD9 | 1: 1000 | ab92726 | Abcam, UK |
| CD63 | 1: 1000 | ab216130 | Abcam, UK |
| TSG101 | 1: 1000 | ab125011 | Abcam, UK |
| Calnexin | 1: 1000 | ab133615 | Abcam, UK |

**Table S6** T2DM-related lncRNAs obtained from the MNDR database

| ncRNA Symbol | ncRNA Category | Species | Disease Name | Score |
| --- | --- | --- | --- | --- |
| MALAT1 | lncRNA | Homo sapiens | T2DM | 1 |
| NONRATT021972 | lncRNA | Homo sapiens | T2DM | 0.999999 |
| GAS5 | lncRNA | Homo sapiens | T2DM | 0.999141 |
| PVT1 | lncRNA | Homo sapiens | T2DM | 0.973352 |
| LINC01370 | lncRNA | Homo sapiens | T2DM | 0.795006 |
| CDKAL1 | lncRNA | Homo sapiens | T2DM | 0.694506 |
| IGF2-AS | lncRNA | Homo sapiens | T2DM | 0.694506 |
| lncRNA-p3134 | lncRNA | Homo sapiens | T2DM | 0.694506 |
| SOCS1 | lncRNA | Homo sapiens | T2DM | 0.694506 |
| uc.48+ | lncRNA | Homo sapiens | T2DM | 0.694506 |
| WFDC21P | lncRNA | Homo sapiens | T2DM | 0.694506 |
